# Parental, child and socio-contextual factors associated with parenting self-efficacy among parents of children aged 0–7 years old: the CIKEO study

**DOI:** 10.1007/s00127-021-02161-2

**Published:** 2021-08-21

**Authors:** Yuan Fang, Amy van Grieken, Irene N. Fierloos, Dafna A. Windhorst, Harrie Jonkman, Clemens M. H. Hosman, Matty R. Crone, Wilma Jansen, Hein Raat

**Affiliations:** 1 Department of Public Health, Erasmus University Medical Centre, Rotterdam, PO Box 2040, 3000 CA Rotterdam, The Netherlands; 2 Department of Cognitive Neuroscience, Donders Institute for Brain, Cognition and Behavior, Radboud University Medical Center, Nijmegen, The Netherlands; 3 TNO Child Health, Leiden, The Netherlands; 4 Verwey-Jonker Institute, Utrecht, The Netherlands; 5 Department of Health Promotion, Maastricht University, Maastricht, The Netherlands; 6 Department of Clinical Psychology, Radboud University, Nijmegen, The Netherlands; 7 Hosman Prevention and Innovation Consultancy, Berg en Dal, The Netherlands; 8 Department of Public Health and Primary Care, Leiden University Medical Center, Leiden, The Netherlands; 9 Municipality of Rotterdam, Rotterdam, The Netherlands

**Keywords:** Family functioning, Fathers, Mothers, Parenting self-efficacy, Risk factors, Protective factors

## Abstract

**Background:**

A high parenting self-efficacy (PSE) has been associated with positive parenting and positive child development. However, there is limited and inconsistent information on factors associated with PSE.

**Objective:**

To investigate factors associated with PSE in parents of children aged 0–7 years old, and to explore whether the associations were different between mothers and fathers.

**Methods:**

We performed a cross-sectional analysis of the baseline data from a prospective cohort study: the CIKEO study. A total of 1012 parents (mean age = 33.8, SD = 5.0) completed self-reported measure of PSE and 18 potential factors associated with PSE.

**Results:**

Multivariable models revealed that lower parenting stress, fewer child behavior problems, better eating behavior, better parental and child general health, a smaller number of children living in the household, higher perceived level of social support and having a migration background were associated with higher levels of PSE (*p* < 0.05). The association between family functioning and PSE differed between mothers and fathers (*p* for interaction = 0.003): with beta and 95% confidence interval being: 1.29 (− 2.05, 0.87), and 0.23 (− 0.46, 3.29), respectively.

**Conclusions:**

A range of parental, child and social-contextual factors in relation to PSE were identified. The patterns of associations for most of the factors were similar among mothers and fathers. However, the association between family functioning and PSE might differ for mothers and fathers. Our findings are relevant for tailoring and implementing successful interventions and effective policy making in child care.

**Trial registration:**

Netherlands National Trial Register number NL7342. Date of registration: 05-November-2018, retrospectively registered.

**Supplementary Information:**

The online version contains supplementary material available at 10.1007/s00127-021-02161-2.

## Introduction

Parenting self-efficacy (PSE) refers to parents’ beliefs or the judgements a parent holds regarding their capabilities to organize and perform parenting tasks [1]. PSE has been linked to a number of parental and child outcomes, including parenting practices, parent and child relationship, parental mental health, and child development [2–5].

In contrast to the well-established associations between PSE and various parenting and child health outcomes, information on factors that determine PSE is limited [2, 6]. Existing studies yield inconsistent results (see Fang et al. [6] for a systematic review). For example, both negative, null and positive associations have been reported between the factors parental sex, age, and ethnic-background and PSE [7–11]. Moreover, studies have focused mainly on high-risk populations (e.g., clinical populations) and on mothers; studies in community samples or fathers are relatively scarce [2, 12]. Also, few studies have compared whether factors are differentially associated with PSE for mothers and fathers, though literature suggest that PSE varies among men and women [9, 13, 14]. For example, difficult child temperament, child behavior problems and family functioning have been suggested to be associated with maternal PSE only [13–15], whereas the effect of parenting stress on PSE might be more notable among fathers [9]. Therefore, expanding knowledge of the factors associated with PSE can be beneficial for the development and implementation of interventions to support parenting behavior and therewith child health outcomes.

In this study, Belsky’s process model [16] of parenting is applied to study factors associated with PSE. According to this model, PSE is affected by parental factors (e.g., parental personality, psychological health), child factors (e.g., temperament) and socio-contextual factors (e.g., experience with children, family composition and perceived support from social network). The aim of this study is to evaluate the association between parental, child and socio-contextual factors and PSE among a community sample of parents of children aged 0–7 years old in. Additionally, we explored whether the associations differed between mothers and fathers. We hypothesized that parental, child and their socio-cultural environment are associated with PSE. Based on previous literature [9, 13–15], we also hypothesized that the associations between socio-demographic characteristics (i.e., migration background, educational level and household income), child age, sex, child behavior problems, family functioning, parenting stress and PSE could be different for mothers and fathers.

## Methods

### Study design and study population

In this study, a cross-sectional design was applied using baseline data of the CIKEO study. The CIKEO (Consortium Integration Knowledge promotion Effectiveness Of parenting interventions in the Netherlands) study is a community-based study with a baseline and a follow-up measurement [17]. The CIKEO study aimed to investigate the use of (elements of) parenting support and the associations between parenting support and outcomes regarding parenting, family functioning and child development. Details of the CIKEO study have been previously published [17]. In brief, parents/caregivers with at least one child up to 7 years old were recruited between October 2017 and December 2019. Participants were recruited in two parts. Participants in Part A were recruited by two regional preventive youth healthcare organizations in the region of Rotterdam (CJG Rijnmond) and Dordrecht (RIVAS Zorggroep). Participants in Part B were recruited by providers of parenting programs or through advertisements on websites about parenting. All invited parents/caregivers received project information, an informed consent form and a baseline questionnaire. The parent/caregiver who spent most time with the child was asked to complete the questionnaire. Parents who provided written informed consent and completed a baseline questionnaire were included. A follow-up measurement was conducted after 12 months of enrollment using questionnaires.

A total of 1118 parents responded to the baseline survey. Questionnaires were excluded when they were completed by other caregivers than parents (*n* = 36), when it was unclear for which child it was completed (*n* = 22), and when there were missing data on the outcome measurement (*n* = 48), leaving a population of 1012 parents for analysis (Supplementary Fig. 1. Compared to parents eligible for analyses (*n* = 1012), parents excluded for analyses (*n* = 106) had a lower education level (*p* < 0.05). There were no differences between the groups regarding other socio-demographic characteristics (Supplementary Table 1).

### Parenting self-efficacy

Parenting self-efficacy was assessed with the Efficacy subscale of the Parenting Sense of Competence Scale [18]. The seven-item subscale has been widely used to explore parental perceived abilities to deal with the demands of parenting among parents of children aged 0–18 years old [18–20]. An example item is “My mother/father was better prepared to be a good mother/father than I am”. Parents indicate their agreement with each item by scoring from 1 (strongly disagree) to 6 (strongly agree). A higher score indicates higher PSE. Score on each item was then summed to create a total score which ranges from 6 to 42. Cronbach’s α for this subscale in our sample was 0.78, which is comparable to that reported in previous studies [18–20].

### Factors associated with PSE

Potential factors were organized and classified into three blocks according to the process model of parenting [16]: parental factors, child factors and socio-contextual factors. Data was obtained from a parent-reported questionnaire.

#### Parental factors

Parental factors included parental age (years) and sex (man/woman), migration background (yes/no), parenting stress, psychological distress and general health status. Migration background was assessed by country of birth of the responding parents and his/her parents (i.e., grandparents of the child): when either the responding parent or one or both of his/her parents was born outside the Netherlands, this was categorized as having a migration background [21].

Parenting stress was assessed with the Parenting Daily Hassles (PDH) [22]. This measure consists of 20 items reflecting the frequency and the intensity of certain stressors. The subscale scores were calculated by adding the scores of the items that belong to each subscale. For the purpose of this study, the intensity subscale was used. Inter-item consistency in our sample was *α* = 0.84. Psychological distress was measured by the Brief Symptom Inventory 18 (BSI-18) scale, an 18-item scale with three subscales [23]. The item 'thoughts of ending your life’ was removed to avoid invasiveness to participants. Inter-item consistency in our sample was *α* = 0.87. For the current analysis, the global severity index (GSI) was computed by summing the score for all the items. For all abovementioned measures, a higher score indicated more stress or distress. General health status was assessed using the first item of the short form 12 health survey (SF-12) [24]; higher scores suggested better general health.

#### Child factors

Child factors included sex (boy/girl), age (years), general health status, parents’ rating on the child’s crying, sleeping, eating behavior and behavior problems. The general health of the child was assessed with the first item of the Child Health Questionnaire [25]. Parent perception of child behavior problems was assessed by the 99-item Child Behavior Checklist (CBCL 1.5–5 years) [26]. Each item received a three-point scale with 0 (not true), 1 (somewhat or sometimes true) and 2 (very true or often true). A total problem score was computed by summing all 99 items; a higher score suggested more problems. The internal consistency of this scale in our sample was 0.95.

Crying behavior was assessed by a question on the extent to which their child’s crying was a problem to parents, with a 5-point response scale (1 = totally agree, 5 = totally disagree). Sleeping and eating behavior was rated by parents on a 10-point scale ranging from 1 (‘worst’) to 10 (‘best’). For these measures, items were coded reversely. Higher scores indicated more problems.

#### Socio-contextual factors

Socio-contextual factors included parental educational level, employment status (no paid, part-time and full time), family composition (one-parent/two-parent family), household income, number of children in the household, family functioning and perceived social support.

Parental educational level was classified into “low” (no education, primary school/primary education/preparatory secondary vocational education), “middle” (senior general secondary education, pre-university education and senior, secondary vocational education) and “high” (higher vocational education/University). Employment status was classified into: no paid job, part-time job (< 36 h per week), full-time job (≥ 36 h per week). Net monthly household income classified into three categories: low (< €2400), middle (€ 2400–5200) and high (> €5200). Family functioning was assessed with the seventh subscale (i.e. General Functioning) of the Family Assessment Device Scale [27]. The 12-item General Functioning scale provides a measure of overall health/pathology of the family by assessing the support and stress within the family. Negatively worded items were reversed and each item received a score range from 1 (best functioning) to 4 (worst functioning). An average score was then calculated for analyses. Higher scores indicate poorer family functioning. The internal consistency of this subscale was 0.87 in our study sample.

Perceived social support was measured using the 12-item Multidimensional Scale of Perceived Social Support scale [28]. Each item received a score ranging from 1 (strongly disagree) to 7 (strongly agree). A total score was calculated by averaging the sum score of 12 items, with a higher score indicates a higher level of perceived support. The internal consistency of this scale was 0.92 in our sample.

### Data analysis

First, differences between mothers and fathers at baseline were assessed using *t*-tests or ANOVA tests for continuous variables and the *χ*^2^ test for categorical variables.

Second, we examine the association between factors and PSE using uni- and multi- variate linear regression models. Variables were entered in the model as blocks according to Belsky’s process model [16], correcting for other variables within this block. Parental factors entered first (model 1), followed by child factors (model 2) and socio-contextual factors (model 3). Finally, a full model (model 4) with all factors was fitted. Variance inflation factors (VIF) were performed to test multi-collinearity of the factors; none of the variables was highly correlated (VIF < 3).

Finally, effect modification by parent sex was examined by adding interaction terms between parent sex and socio-demographic characteristics (i.e., migration background, educational level and household income), parenting stress, child age, sex and behavior problems, family functioning to the multivariable model (model 4) separately [9, 10, 13, 14]. Considering the relatively small number of fathers in our study, a penalized regression model [29] was fitted via the ‘glmnet’ package in R to get the coefficients for mothers and fathers separately if the interaction term was significant (*p* < 0.10) [30]. Bootstrapping with 1000 iterations was used to obtain the 95% confidence interval (CI) of the estimated coefficients.

Some variables had missing data, ranging from 0.001% (parental age) to 6.3% (household income). Multiple imputation was applied to all variables included in this study using the R package ‘mice’. Five imputed datasets were generated, and pooled to estimate the β and 95% CI.

All statistical analyses were performed using R version 3.6.2. All tests were two-sided and a *p* value < 0.05 was considered as significant.

## Results

### Sample characteristics

Table 1 presents the characteristics of the sample. Our study population consisted of 938 mothers and 74 fathers of 0 to 7-year-old children; the mean child age was 3.2 years (SD = 1.9), and 48.3% were girls. The mean age of the parents was 34.1 years (SD = 5.1), fathers were on average older than mothers [38.1(6.1) vs 33.8(4.9), *p* < 0.001]. Responding parents were highly educated (49%), employed (95.7%), living with a partner (93%), had a middle or high income (84.5%), had two or more children living in the household (68.6%) and had no migration background (85.7%). Compared to fathers, mothers were more likely to be involved in part-time jobs (16.2% vs 74.4%, *p* < 0.001).

**Table 1:** Descriptive characteristics of the study population (*n* = 1012)

Variables	Missing *n* (%)	Total (*n* = 1012)	Mothers (*n* = 938)	Fathers (*n* = 74)	*p* value^†^
Parental factors					
Age of the respondent (years), mean (SD)	1 (0.1)	34.1 (5.1)	33.8 (4.9)	38.1 (6.1)	< 0.001
Partner’ age (years), mean (SD)	31 (3.1)	36.2 (5.7)	36.2 (5.7)	35.6 (4.9)	0.39
Migration background of the respondent (yes, %)	20 (2)	164 (16.5)	139 (15.1)	25 (33.8)	< 0.001
General health of the respondent, mean (SD)	8 (0.8)	69.5 (20.0)	69.5 (19.7)	68.8 (24.1)	0.77
Psychological distress of the respondent, mean (SD)	18 (1.8)	5.5 (6.5)	5.4 (6.4)	6.7 (8.0)	0.09
Parenting Stress of the respondent, mean (SD)	53 (5.2)	27.1 (10.0)	27.2 (10.0)	25.9 (10.0)	0.29
Parenting Self-efficacy of the respondent, mean (SD)	0 (0)	31.9 (4.3)	31.9 (4.3)	31.0 (5.1)	0.08
Child factors					
Child age (years), mean (SD)	8 (0.8)	3.2 (1.9)	3.2 (1.9)	3.7 (2.1)	0.06
Sex (Girls%)	6 (0.6)	486 (48.3)	456 (48.8)	30 (41.7)	0.29
General health, mean (SD)	13 (1.3)	79.0 (16.5)	78.8 (16.2)	81.4 (20.7)	0.2
Child behavior health, mean (SD)^‡^	27 (2.7)	20.5 (16.9)	20.4 (16.6)	21.7 (20.5)	0.54
Sleeping Score, mean (SD)^‡^	19 (1.9)	3.8(1.5)	3.8 (1.5)	3.9 (1.5)	0.54
Eating Score, mean (SD)^‡^	16 (1.6)	3.3 (1.6)	3.3 (1.5)	3.7 (1.9)	0.06
Crying Score, mean (SD)^‡^	16 (1.6)	4.5 (0.7)	4.5 (0.7)	4.5 (0.8)	0.59
Socio-contextual factors					
Parental educational level	2 (0.2)				0.03
Low (%)		77 (7.6)	72 (7.7)	5 (6.8)	
Middle (%)		372 (36.8)	355 (37.9)	17 (23.0)	
High (%)		561 (55.5)	509 (54.4)	52 (70.3)	
Partners’ educational level	38 (3.8)				0.004
Low (%)		134 (13.8)	126 (14.0)	8 (11.0)	
Middle (%)		380 (39.0)	363 (40.3)	17 (23.3)	
High (%)		460 (47.2)	412 (45.7)	48 (65.8)	
Employment status	1 (0.1)				< 0.001
No paid job (%)		184 (18.2)	171 (18.2)	13 (17.6)	
Part time job (%)		709 (70.1)	697 (74.4)	12 (16.2)	
Full time job (%)		118 (11.7)	69 (7.4)	49 (66.2)	
Partners’ employment status	27 (2.7)				< 0.001
No paid job (%)		42 (4.3)	30 (3.3)	12 (16.4)	
Part time job (%)		147 (14.9)	97 (10.6)	50 (68.5)	
Full time job (%)		796 (80.8)	785 (86.1)	11 (15.1)	
Net household income (per month)	64 (6.3)				0.38
Low (< 2400)		150 (15.8)	140 (16.0)	10 (14.1)	
Middle (2400–5200)		686 (72.4)	637 (72.6)	49 (69.0)	
High (> 5200)		112 (11.8)	100 (11.4)	12 (16.9)	
Number of children in household	0 (0)				0.24
1 (%)		318 (31.4)	287 (30.6)	31 (41.9)	
2 (%)		450 (44.5)	421 (44.9)	29 (39.2)	
3 (%)		171 (16.9)	160 (17.1)	11 (14.9)	
≥ 4 (%)		73 (7.2)	70 (7.5)	3 (4.1)	
Family composition (one-parent, %)	4 (0.4)	69 (6.8)	65 (7.0)	4 (5.5)	0.81
Family functioning, mean (SD)^‡^	17 (1.7)	1.4 (0.4)	1.4 (0.4)	1.6 (0.4)	0.004
Perceived Social Support, mean (SD)	8 (0.8)	5.9 (0.9)	5.9 (0.9)	5.4 (1.0)	< 0.001

This table is based on the non-imputed dataset

*SD *standard deviation

^†^*P *values are calculated by *χ*^2^ test for categorical variables and *t*-test/ANOVA for continuous variables. ^‡^Higher scores indicate more problems or worse family functioning

Mothers reported higher perceived social support than fathers [5.9 (0.9) vs 5.4 (1.0), *p* < 0.001], while fathers reported better family functioning [1.4 (0.4) vs 1.6 (0.4), *p* = 0.004]. The mean PSE score for mothers and fathers was 31.9 (4.3) and 31.0 (5.1), respectively (*p* = 0.08).

### Factors associated with PSE

The association between various parental, child and socio-contextual factors and PSE is shown in Table 2. The final model (model 4) was statistically significant (*F* = 21.56, *p* < 0.05) and accounted for 28% of the variance in PSE. The block of parental factors accounted for most of the variance (20%) in PSE score, followed by child characteristics (16%) and social-contextual characteristics (12%).

**Table 2:** Associations of parental, child and socio-contextual characteristics with parental self-efficacy in parents of a child aged 0–7 years old (*N* = 1012)

Variables	Model1 β (95%CI)	Model2 β (95%CI)		Model3 β (95%CI)	Model4 β (95%CI)
Parental factors					
Gender (ref: fathers)	***1.15 (0.14, 2.16)***				1.01 ( − 0.17, 2.2)
Age of the respondent (older)	0.04 ( − 0.03, 0.12)				0.03 (− 0.04, 0.10)
Partners’ age (older)	0.02 ( − 0.05, 0.08)				0.04 (− 0.02, 0.10)
Migration background of the respondent (yes)	***0.75 (0.05, 1.44)***				***0.90 (0.23, 1.56)***
General health of the respondent (better)	***0.03 (0.02, 0.04)***				***0.02 (0.003, 0.03)***
Psychological distress of the respondent (higher)^†^	***− 0.09 ( − 0.14, − 0.05)***				− 0.04 (− 0.08, 0.01)
Daily parenting stress of the respondent (higher)^†^	***− 0.13 ( − 0.16, − 0.11)***				***− 0.08 (− 0.11, − 0.05)***
Child factors					
Child age (older)		− 0.11 ( − 0.24, 0.02)			− 0.08 (− 0.22, 0.06)
Child sex (ref: boys)		0.13 ( − 0.37, 0.62)			0.32 (− 0.15, 0.79)
General health (better)		***0.04 (0.02, 0.05)***			***0.02 (0.004, 0.04)***
Child behavior problem(more)^†^		***− 0.06 ( − 0.08, − 0.04)***			***− 0.02 (− 0.04, − 0.01)***
Sleeping problem (more)^†^		− 0.12 (− 0.31, 0.07)			0.15 (− 0.03, 0.32)
Eating problem(more)^†^		***− 0.24 (− 0.41, − 0.07)***			***− 0.17 (− 0.34, − 0.01)***
Crying problem(more)^†^		***− 0.43 (− 0.74, − 0.12)***			− 0.28 (− 0.57, 0.02)
Socio-contextual factors					
Family composition (one-parent)			0.43 (− 0.80, 1.66)		− 0.33 (− 1.53, 0.87)
Number of children (more)			***− 0.41 (− 0.69, − 0.14)***		***− 0.46 (− 0.74, − 0.17)***
Perceived social support (more)			***0.80 (0.48, 1.12)***		***0.40 (0.10, 0.70)***
Family functioning (worse)^†^			***− 2.57 (− 3.29, − 1.84)***		***− 1.36 (− 2.06, − 0.65)***
Employment status (ref: not paid)					
Part time job			− 0.47 (− 1.2, 0.26)		− 0.22 (− 0.91, 0.47)
Full time job			− 0.02 (− 1.06, 1.03)		− 0.04 (− 1.05, 0.96)
Partners’ employment status (ref: not paid)					
Part time job			− 0.47 (− 1.92, 0.97)		− 0.39 (− 1.71, 0.94)
Full time job			− 0.26 (− 1.61, 1.09)		− 0.29 (− 1.53, 0.94)
Educational level (ref: high)					
Middle			− 0.22 (− 0.79, 0.36)		− 0.09 (− 0.63, 0.44)
Low			− 0.68 (− 1.77, 0.42)		− 0.55 (− 1.56, 0.46)
Partners’ educational level (ref: high)					
Middle			***− 0.76 (− 1.36, − 0.16)***		− 0.61 (− 1.15, − 0.06)
Low			0.57 (− 0.30, 1.45)		0.51(− 0.30, 1.31)
Household income (ref: high)					
Middle			− 0.73 (− 1.59, 0.14)		− 0.20 (− 0.99, 0.59)
Low			− 0.55 (− 1.76, 0.66)		0.23 (− 0.91, 1.38)
Recruitment method (ref: method A)					− 0.54 (− 1.33, 0.26)
*R*^2^ (adjusted)	0.20	0.16	0.12		0.28

This table is based on the imputed dataset. Bold and Italic print indicates statistical significance at 0.05 level. Values represent were coefficient and 95% confidence intervals derived from multiple regression analyses

*ref* reference

^†^Higher score indicates more problems

For parental factors*,* lower levels of parenting stress, better general health and having a migration background were independently associated with higher levels of PSE (*p* < 0.05). Parental gender, age and distress were not found to be associated with PSE (*p* > 0.05).

Of the child factors better general health, less behavior problems as well as less eating problems were independently associated with higher PSE (*p* < 0.05). No associations were found between child age, sex, sleeping/ crying problems and PSE (*p* > 0.05).

Regarding socio-contextual factors, parents who had fewer children living in the house, parents who perceived higher levels of social support and with better family functioning, had higher PSE scores (*p* < 0.05). No significant associations were observed between other social-contextual factors including household income, educational level and employment status and PSE (*p* > 0.05).

### Differences between subgroup mothers and fathers

Results from the interaction analyses showed no statistically significant sex difference in the associations between selected factors and PSE (*p* > 0.10), except for family functioning (*p* for interaction = 0.003). Better family functioning was statistically significantly associated with higher PSE in the subgroup mothers (beta: − 1.29, 95%CI: − 2.05, 0.87), but not in the subgroup fathers (beta: 0.23, 95%CI: − 0.46, 3.29) (Supplementary Table2).

## Discussion

In this study, factors associated with parenting self-efficacy was evaluated in a community sample of parents with a child between 0 and 7 years old. Associations were observed between several parental (i.e., parenting stress, general health status, migration background), child (i.e., eating and behavior problems, general health status) and social-contextual factors (i.e., number of children, perceived social support) and PSE. Family functioning was associated with higher maternal rather than paternal PSE.

### Parental factors and PSE

Three parenting factors were significantly associated with PSE: having a migration background, reporting less parenting stress, and reporting a better general health status.

Having a migration background was associated with higher PSE in our sample. Studies in other countries have reported differences in PSE among subgroups with different ethnic backgrounds. For instance, a study in America observed that black mothers reported higher PSE than mothers with other ethnic backgrounds [11]. What is known, is that parents with different cultural backgrounds could have different attributions, attitudes and beliefs in parenting [31]. These differences may buffer potential negative effects of acculturation conflicts on perceived parenting competence [31, 32]. More studies are needed across countries to study the association between cultural background and PSE, and potential pathways underlying this association [6].

The finding that higher parenting stress is related to decreased PSE is in line with the finding reported in a recent systematic review [6]. Belsky’s process model has indicated that parental psychological status is a key variable in parenting; parenting stress may influence parenting directly, and parents’ social relationships indirectly, which may subsequently undermine PSE [11].

The association of a better health status with higher levels of PSE, is consistent with the study of Giallo [15], but inconsistent with other studies [8, 33]. It is likely that parents experiencing health problems may find parenting more demanding, more difficult to engage in daily child-rearing activities and more challenging to meet the child’s needs [8, 15]. Subsequently, this may cause increased parenting stress and undermine their confidence as a parent.

### Child factors and PSE

Parents reporting their child having more behavior problems, poorer eating behavior, and a poorer perceived general health status showed lower PSE. An inversed association has been consistently observed for perceived parenting task difficulties and PSE [34–36]. Parenting children who are less healthy and have more behavior problems can require additional energy, skills and support which may directly impair parents’ confidence in child rearing. No associations were observed between child sleeping and crying behavior score and PSE. Sleeping and crying problems are common during the first year of life and are transient [37]. In the current study only a small percentage of children was aged 0–1 years and reported with high sleeping and crying behavior scores (*n* = 98). Our results highlight the importance for child health care providers to pay attention to the potential challenges parents encounter in parenting children with behavior problems.

Child age was not associated with PSE in our study. Findings with regard to the associated between child age and PSE have been mixed [6, 38]. Previous studies have suggested that PSE is dynamic rather than fixed, parents may have to practice new parenting skills to meet the needs of different parenting tasks at different child developmental stages [1, 2]. We included children with a broad age range (i.e., 0–7 years old). We tested the association between child age and PSE in four age groups (i.e., 0–1, 1–3, 3–4 and 4–7 years old) of children respectively and found no association between age and PSE in any of these subgroups (data not shown). However, these analyses were performed in relatively small subgroups thus limiting the power to detect associations. Future longitudinal studies across multiple developmental stages of children are recommended to gain insight in the association between child age and PSE [6].

### Socio-contextual factors and PSE

Having fewer children in the family, a better family functioning and a higher perceived social support was associated with higher PSE, which is in line with earlier studies [7, 36, 39].

According to Bandura, having experience with an activity or behavior is a primary contributor to one’s self-efficacy in a particular domain [1]. Parenting experience, from having more children in the family, might also boost PSE. Contradicting this hypothesis, our results suggested that parents with more children in the house had lower PSE. A recent systematic review found an equivocal association between number of children and PSE in mothers [6]. It’s possible that parents with more children experience limited time and energy for each child, and overall higher levels of family stress [6, 8]. In this situation potentially more negative parenting practices and experiences take place that eventually might lead to undermined parenting self-efficacy [8, 40].

Family is the place where parents and their children spend the most time together. Better family functioning has been linked to better family problem-solving ability, less parenting stress and other positive outcomes in parenting [39]. Instead, undesirable family functioning can be a source of stress for parents and undermine their PSE [16].

A higher level of social support is a well-established predictor of optimal parenting practices and parent wellbeing [36, 39]. Parents can get advice and support on child rearing from their partners, families, friends and social networks. With support and encouragement, parents may develop and maintain PSE easier [39]. Fathers in our sample reported lower level of social support than mothers. A previous study has indicated a gender difference in the perception and the health effects of social support [10]. Additionally, this study reported that man are also less likely to seek social support [41]. Hence, health promotion efforts may take these aspects into account whilst supporting families.

Regarding socio-economic status (SES) indictors, parental educational level, employment status and household income were not associated with PSE. These findings are inconsistent with previous studies [10, 11]. Compared to highly educated parents, less educated parents might perceive fewer complexities in parenting, thus are more confident in parenting [10]. However, our study population consisted of high-educated, and relatively high-income families; this may explain the absence of this association in our study. To shed light on the association between SES and PSE, studies with families of diverse backgrounds are needed.

### Mothers, fathers and PSE

Previous studies have suggested that mothers and fathers might perceive their role as a parent differently [9, 13, 14]. In a longitudinal study, Knauth et al. [13] found that maternal PSE rather than paternal PSE was affected by family functioning. In line herewith, higher PSE was associated with more positive family functioning in mothers but not in fathers in our study. An explanation for this sex difference has been suggested by the disparities in the perception, coping and experience of family stress [41, 42]. For instance, Rhoads reported that women are more vulnerable than men to undesirable family environment, e.g. problems in marriage/relationship [42]. Also, Shek [41] indicated that women might have lower coping ability towards these marital and familial problems. However, we included a small number of fathers, who may not be representative to the whole population of fathers in the Netherlands. In general, fathers are underrepresented in parenting-related studies [6]. Our findings highlight the importance of inviting both mothers and fathers to participate in studies but also the difficulty in involving large numbers of fathers in studies. There is an increase in caring fatherhood [43] therefore longitudinal studies with representative samples of men and women are recommended to study the potential sex differences.

### Methodological considerations

One strength of this study is the relatively large sample size and the availability of a large group of potential factors correlated to PSE. Another strength of our study is the inclusion of fathers, which allowed us to perform subgroup analysis and explore the potential differences among mothers and fathers.

However, several limitations should also be addressed. Firstly, causality cannot be inferred given the cross-sectional study design. In this study factors that co-vary with PSE were identified. The construct of PSE is complex with multiple factors that could interplay with each other [16, 40]. Therefore, to increase understanding of the construct of PSE, future studies could use methods such as structural equation modeling to test the effects of specific pathways in determining PSE are recommended [6]. Secondly, we relied on parental self-reported data, which may lead to socially desirable answers. Finally, participants were largely Dutch, highly educated and relatively high-income families. Besides, a small number of fathers participated. These fathers, however, might not be fully representative to the whole population of fathers in the Netherlands. These limitations of the sample should be taken into account in the interpretation of findings. The correlates of PSE in parents from diverse cultural and economic groups may differ from the findings of this study. Longitudinal studies involving larger and more diverse study population (e.g., ethnic-minorities, low income/educated parents, men/women) are recommended.

## Conclusion

In conclusion, a range of parental, child and socio-contextual factors associated with PSE were identified. Higher parenting stress, inferior parental or child general health status, more child eating- and behavior problems, more children living in the household, poorer family functioning and lower levels of perceived social support were associated with decreased PSE. A potential difference between mothers and fathers regarding the association between family functioning and PSE was observed. Child health care providers should be aware of the particular needs of parents who experience more than the typical demands of parenting. Our findings are also relevant for the tailoring and implementing of interventions focusing on parenting to support child health and development.

## Supplementary Information

Below is the link to the electronic supplementary material.

Supplementary file1 (DOCX 38 KB)

## Data Availability

The data that support the findings of this study are available from the corresponding author, upon reasonable request.

## Code availability

The code is available from the first author (YF), upon reasonable request.
