# Supplementary material for: Parental, child and socio-contextual factors associated with parenting self-efficacy among parents of children aged 0–7 years old: the CIKEO study
Source: Soc Psychiatry Psychiatr Epidemiol. 2021 Aug 21;57(3):623–32. doi: 10.1007/s00127-021-02161-2 (PMC8934325; doi:10.1007/s00127-021-02161-2)
Supplement: Supplementary file 1 — Supplementary file1 (DOCX 38 KB) [file 127_2021_2161_MOESM1_ESM.docx]

**Supplementary files**

Fang Y., et al. Parental, child and socio-contextual factors associated with parenting self-efficacy among parents of children aged 0-7 years old: the CIKEO study

Parents/Caregivers recruited form the youth health care centers

**N=1118**

Parents of children aged 0-7 years old

**N=1082**

N=36

Excluded: Filled by other caregivers

Parents of children aged 0-7 years old

**N=1060**

Population for analysis

**N=1012**

Mothers: N = 938

Fathers: N =74

N=22

Excluded: unclear for which child it was completed

N=48

Excluded: Missing data on parenting self-efficacy

**Supplementary Figure 1.** Flowchart of participants included for analysis

| **Supplementary Table 1. Non response analysis** | | | | | | | | | | | | | | | | | |
| --- | --- | --- | --- | --- | --- | --- | --- | --- | --- | --- | --- | --- | --- | --- | --- | --- | --- |
|  |  | | Excluded for analysis | | | | | Included for analysis | | | | | | | P value | | |
|  |  |  | n=106 | | | | | n=1012 | | | | | | |  |  |  |
| **Parental Factors** | | |  | | | | |  | | | | | | |  | | |
| Age of the respondent (years), mean (SD) | | | 34.8 (5.9) | | | | | 34.1 (5.1) | | | | | | | 0.23 | | |
| Partner' age of the respondent (years), mean (SD) | | | 35.3 (6.2) | | | | | 36.2 (5.7) | | | | | | | 0.14 | | |
| General health of the respondent, mean (SD) | | | 64.8 (21.9) | | | | | 69.5 (20.0) | | | | | | | 0.07 | | |
| Ethnic background (Non-Dutch%) | | | 21 (20.6) | | | | | 164 (16.5) | | | | | | | 0.37 | | |
| Parenting Self-efficacy of the respondent, mean (SD) | | | 32.2(4.1) | | | | | 31.9(4.3) | | | | | | | 0.54 | | |
| parenting distress of the respondent, mean (SD) | | | 5.5 (6.6) | | | | | 5.5 (6.5) | | | | | | | 0.96 | | |
| Parenting stress of the respondent, mean (SD) | | | 27.1 (10.7) | | | | | 27.1 (10.0) | | | | | | | 1 | | |
| **Child Factors** | | |  | | | | | | | | | | | |  | | |
| Child age (years), mean (SD) | | | 3.3 (2.1) | | | | | 3.2 (1.9) | | | | | | | 0.9 | | |
| Sex (Girls%) | | | 52 (50.0) | | | | | 486 (48.3) | | | | | | | 0.82 | | |
| General health, mean (SD) | | | 77.0 (17.7) | | | | | 79.0 (16.5) | | | | | | | 0.35 | | |
| Child behavior health, mean (SD) | | | 19.4 (13.8) | | | | | 20.5 (16.9) | | | | | | | 0.63 | | |
| Sleeping Score, mean (SD) | | | 4.2 (1.7) | | | | | 3.8 (1.5) | | | | | | | 0.08 | | |
| Eating Score, mean (SD) | | | 3.4 (1.5) | | | | | 3.3 (1.6) | | | | | | | 0.87 | | |
| Crying Score, mean (SD) | | | 1.5 (0.6) | | | | | 1.5 (0.7) | | | | | | | 0.77 | | |
| **Socio-contextual Characteristics** | | |  | | | | |  | | | | | | |  | | |
| Family composition (one-parent, %) | | | 6 (5.8) | | | | | 69 (6.8) | | | | | | | 0.83 | | |
| Family functioning, mean (SD) ^‡^ | | | 1.5 (0.5) | | | | | 1.4 (0.4) | | | | | | | 0.32 | | |
| Perceived Social Support, mean (SD) | | | 5.7 (1.1) | | | | | 5.9 (0.9) | | | | | | | 0.1 | | |
| Educational Level | | |  | | | | |  | | | | | | | 0.03 | | |
|  | Low (%) | | 13 (12.6) | | | | | 77 (7.6) | | | | | | |  | | |
|  | Middle (%) | | 46 (44.7) | | | | | 372 (36.8) | | | | | | |  | | |
|  | High (%) | | 44 (42.7) | | | | | 561 (55.5) | | | | | | |  | | |
| Partners' Educational Level | | |  | | | | |  | | | | | | | 0.84 | | |
|  | Low (%) | | 13 (13.0) | | | | | 134 (13.8) | | | | | | |  | | |
|  | Middle (%) | | 42 (42.0) | | | | | 380 (39.0) | | | | | | |  | | |
|  | High (%) | | 45 (45.0) | | | | | 460 (47.2) | | | | | | |  | | |
| Employment Status | | |  | | | | |  | | | | | | | 0.13 | | |
|  | No paid job (%) | | 27 (26.2) | | | | | 184 (18.2) | | | | | | |  | | |
|  | Part time job (%) | | 64 (62.1) | | | | | 709 (70.1) | | | | | | |  | | |
|  | Full time job (%) | | 12 (11.7) | | | | | 118 (11.7) | | | | | | |  | | |
| Partner' Employment Status | | |  | | | | |  | | | | | | | 0.95 | | |
|  | No paid job (%) | | 4 (4.0) | | | | | 42 (4.3) | | | | | | |  | | |
|  | Part time job (%) | | 14 (13.9) | | | | | 147 (14.9) | | | | | | |  | | |
|  | Full time job (%) | | 83 (82.2) | | | | | 796 (80.8) | | | | | | |  | | |
| Net Household Income (per month) | | |  | | | | |  | | | | | | | 0.64 | | |
|  | Low (<2400) | | 18 (18.2) | | | | | 150 (15.8) | | | | | | |  | | |
|  | Middle (2400-5200) | | 72 (72.7) | | | | | 686 (72.4) | | | | | | |  | | |
|  | High (>5200) | | 9 (9.1) | | | | | 112 (11.8) | | | | | | |  | | |
| Number of children in household | | |  | | | | |  | | | | | | | 0.37 | | |
|  | 1(%) | | 33 (31.1) | | | | | 318 (31.4) | | | | | | |  | | |
|  | 2(%) | | 45 (42.5) | | | | | 450 (44.5) | | | | | | |  | | |
|  | 3(%) | | 16 (15.1) | | | | | 171 (16.9) | | | | | | |  | | |
|  | ≥4(%) | | 12 (11.3) | | | | | 73 (7.2) | | | | | | |  | | |
| Table is based on the non-imputed dataset. ^†^ P-values are calculated by Chi-square test for categorical variables and t-test/ANOVA for continuous variables. ^‡^ higher scores indicate more problems. | | | | | | | | | | | | | | | | | |
| **Supplementary Table 2**. Coefficient estimates and 95% confidence interval for ridge regression in mothers and fathers | | | | | | | | | | | | | | | | | |
|  | | **Mothers (n=938)** | | | | |  | | **Fathers (n=74)** | | | | |  |  |  |  |
|  | | **beta coefficient ^*^** | | **Bootstrapped 95%CI ^#^** | | |  | | **beta coefficient** | | **Bootstrapped 95%CI** | | |  |  |  |  |
| **Parental Factors** | | |  | |  | | |  | |  | |  | | |  |  |  |
| Age of the respondent (older) | | 0.01 | | -0.03, 0.07 | | |  | | 0.01 | | -0.13, 0.16 | | |  |  |  |  |
| Partners' age (older) | | 0.02 | | -0.01, 0.07 | | |  | | -0.001 | | -0.16, 0.11 | | |  |  |  |  |
| Migration background of the respondent (yes) | | **0.51** | | **0.04, 1.11** | | |  | | 0.52 | | -0.20, 2.83 | | |  |  |  |  |
| General health of the respondent (better) | | **0.01** | | **0.004, 0.02** | | |  | | 0.01 | | -0.02, 0.04 | | |  |  |  |  |
| Psychological distress of the respondent (higher) ^†^ | | **-0.05** | | **-0.09, -0.02** | | |  | | -0.01 | | -0.10, 0.10 | | |  |  |  |  |
| Daily parenting stress of the respondent (higher) ^†^ | | **-0.07** | | **-0.09, -0.05** | | |  | | -0.02 | | -0.11, 0.03 | | |  |  |  |  |
| **Child Factors** | | |  | |  | | |  | |  | |  | | |  |  |  |
| Child age (older) | | -0.06 | | -0.15, 0.03 | | |  | | -0.10 | | -0.50, 0.16 | | |  |  |  |  |
| Child sex (ref: boys) | | 0.18 | | -0.14, 0.60 | | |  | | 0.20 | | -1.35, 1.51 | | |  |  |  |  |
| General health (better) | | 0.02 | | 0.003, 0.03 | | |  | | 0.02 | | -0.02, 0.04 | | |  |  |  |  |
| Child behavior problem(more) ^†^ | | **-0.02** | | **-0.03, -0.01** | | |  | | -0.02 | | -0.09, 0.003 | | |  |  |  |  |
| Sleeping problem(more) ^†^ | | -0.14 | | -0.26, 0.00 | | |  | | -0.07 | | -0.37,0.39 | | |  |  |  |  |
| Eating problem (more) ^†^ | | **-0.15** | | **-0.29, -0.03** | | |  | | -0.13 | | -0.60,0.18 | | |  |  |  |  |
| Crying problem(more) ^†^ | | -0.41 | | -0.84, 0.00 | | |  | | -0.47 | | -1.48, 0.11 | | |  |  |  |  |
| **Socio-contextual Factors** | | |  | |  | | |  | |  | |  | | |  |  |  |
| Family composition (one-parent) | | 0.07 | | -0.87, 1.04 | | |  | | 0.06 | | -4.40, 3.07 | | |  |  |  |  |
| Number of children (more) | | -0.29 | | -0.53, -0.15 | | |  | | -0.18 | | -1.01, 0.74 | | |  |  |  |  |
| Perceived social support (more) | | **0.28** | | **0.03, 0.52** | | |  | | **0.43** | | **0.18, 1.68** | | |  |  |  |  |
| Family functioning (worse) ^†^ | | **-1.29** | | **-2.05, -0.87** | | |  | | 0.23 | | -0.46, 3.29 | | |  |  |  |  |
| Employment Status of the respondent (ref: not paid) | |  | |  | | |  | |  | |  | | |  |  |  |  |
| Part time job | | -0.19 | | -0.63, 0.32 | | |  | | 0.06 | | -0.91, 2.79 | | |  |  |  |  |
| Full time job | | 0.14 | | -0.71, 0.88 | | |  | | 0.04 | | -1.26, 2.01 | | |  |  |  |  |
| Partners’ employment Status (ref: not paid) | |  | |  | | |  | |  | |  | | |  |  |  |  |
| Part time job | | 0.14 | | -0.43, 0.88 | | |  | | -0.25 | | -2.41, 0.28 | | |  |  |  |  |
| Full time job | | 0.02 | | -0.53, 0.83 | | |  | | 0.09 | | -1.36, 1.97 | | |  |  |  |  |
| Educational level of the respondent (ref: high) | |  | |  | | |  | |  | |  | | |  |  |  |  |
| middle | | -0.06 | | -0.44, 0.36 | | |  | | -0.18 | | -3.23, 0.59 | | |  |  |  |  |
| low | | -0.27 | | -1.004, 0.39 | | |  | | -1.19 | | -4.95, 1.17 | | |  |  |  |  |
| Partners’ educational level (ref: high) | |  | |  | | |  | |  | |  | | |  |  |  |  |
| middle | | **-0.57** | | **-1.05, -0.25** | | |  | | 0.35 | | -0.52, 2.37 | | |  |  |  |  |
| low | | 0.35 | | -0.13, 0.88 | | |  | | -0.09 | | -1.97, 3.19 | | |  |  |  |  |
| Household income (ref: high) | |  | |  | | |  | |  | |  | | |  |  |  |  |
| middle | | -0.12 | | -0.54, 0.38 | | |  | | -0.35 | | -1.58, 1.44 | | |  |  |  |  |
| low | | 0.03 | | -0.57, 0.65 | | |  | | 0.37 | | -0.58, 2.38 | | |  |  |  |  |
| Recruitment methods | | -0.35 | | -1.06, 0.28 | | |  | | -0.87 | | -3.98, 1.96 | | |  |  |  |  |
| R Square | | 0.280 | |  | | |  | | 0.340 | |  | | |  |  |  |  |
| Notes: * beta coefficients were calculated using penalized (ridge) regression; # 95% confidence intervals were derived from bootstrapping with 1000 iterations. Bold printed numbers indicate a statistical significance beta coefficient at 0.05 level. † Higher score indicates more problems. ref=reference. | | | | | | | | | | | | | | |  |  |  |
